# Treatment of hereditary hypotrichosis simplex of the scalp with oral minoxidil and growth factors

**DOI:** 10.1111/dth.15671

**Published:** 2022-07-04

**Authors:** Marina Vastarella, Fabrizio Martora, Sonia Ocampo‐Garza, Angela Patri, Teresa Battista, Paola Nappa, Gabriella Fabbrocini, Mariateresa Cantelli

**Affiliations:** ^1^ Dermatology Unit, Department of Clinical Medicine and Surgery University of Naples Federico II Naples Italy; ^2^ Departamento de Dermatología, Hospital Universitario Dr. José Eleuterio González Universidad Autónoma de Nuevo León Monterrey Mexico

Dear Editor,

Hypotrichosis simplex (HS) is a rare form of hereditary nonsyndromic alopecia characterized by sparse or absent scalp hair, without other ectodermal or systemic abnormalities.^1^, ^2^ Affected individuals are usually born with normal hair but experience a widespread and progressive loss of scalp hair, which begins in childhood and progresses with age.^1^, ^2^ No treatments are currently available for congenital hypotrichosis.

We report the case of a 14‐year‐old girl who showed an improvement in her alopecia after 3 months of treatment with oral minoxidil. Since birth, the patient presented with progressive hair loss. In addition, since the first year of life she referred short and fragile eyelashes and eyebrows. The patient had normal physical development without other neurological or ectodermal alterations. Family history of any type of alopecia was denied.

Clinically, the patient presented diffuse alopecia with sparse, short, and thin hair. Pull test was positive. Trichological examination highlighted yellow dots and vellus hairs on the whole scalp (Figure 1A,C). Trichogram of the patient's hair showed distorted anagen hair bulbs without abnormalities of the hair shaft.

**FIGURE 1:**
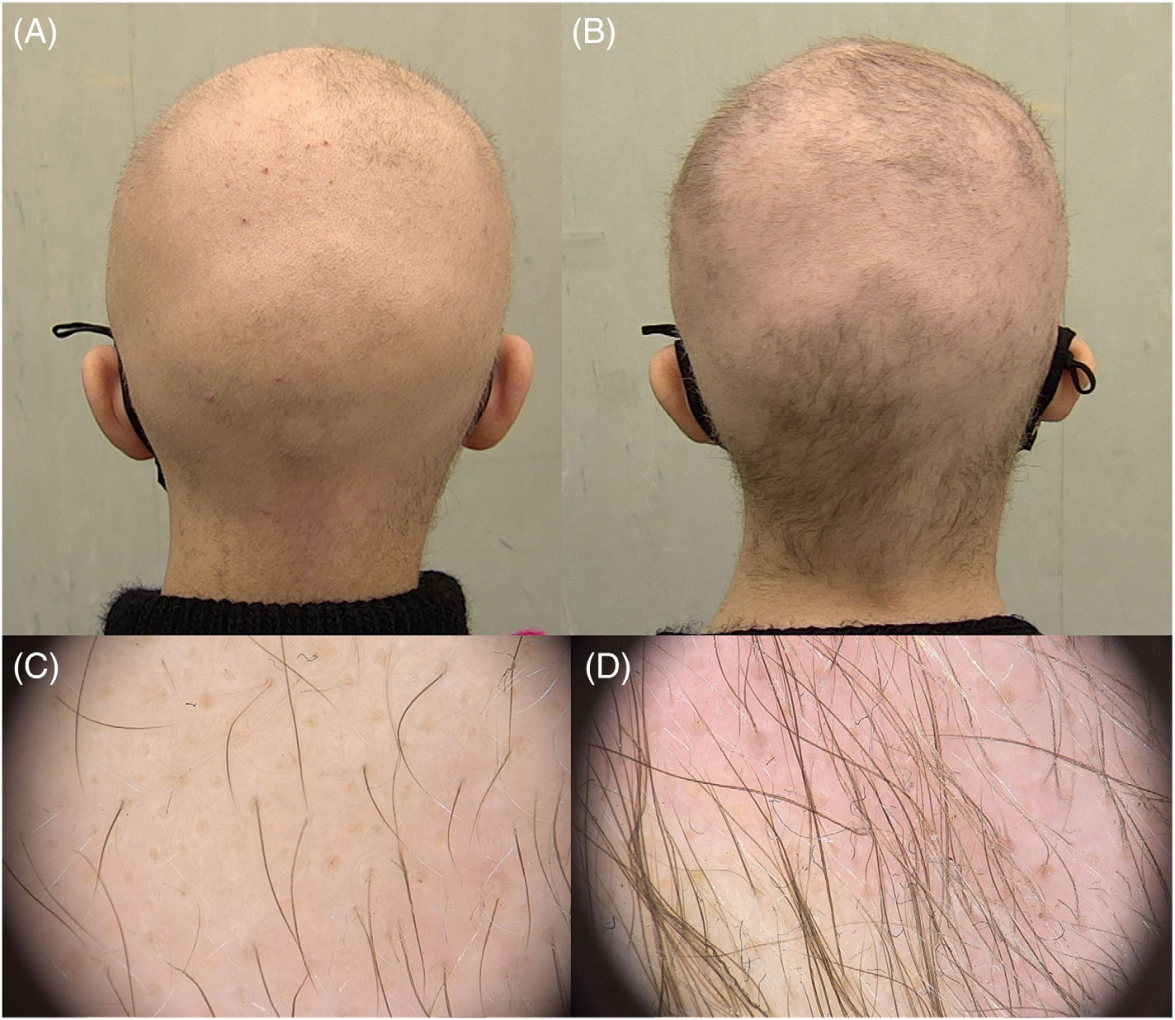
Before (A,C) and 3 months after (B,D) treatment with oral minoxidil 0.25 mg. The clinical and the videotricoscopic evaluation at the follow‐up visit highlighted a significant improvement in hair density (B,D) and hair thickness compared to the baseline

The following analyses were requested: blood count, erythrocyte sedimentation rate, liver, thyroid and renal function tests, serum electrolytes, urinalysis, blood glucose, the glucose tolerance test, Hb A1C, copper, zinc, and ferritin proteins, quantitative immunoglobulins, and amino acids all within normal values.

Given the variety of differential diagnoses, especially in pediatric age with hair shaft disorders with or without fragility,^3^ we performed a punch biopsy. Histological examination showed a marked decrease in hair follicles, surrounded by mild chronic inflammatory infiltrate conformed predominantly of lymphocytes. In addition, we carried out a genetic investigation, which revealed a LIPH mutation, confirming our diagnostic suspicion (woolly hair hypotrichosis 2). The patient underwent a 3‐month cycle with oral minoxidil 0.25 mg/die and growth factors every night (growth factors contained mainly caffeine and hyaluronic acid), clinical and videotricoscopic follow‐up evaluation highlighted a significant improvement in hair density and hair thickness compared to baseline (Figure 1B,D).

HS is a rare form of hereditary nonsyndromic alopecia characterized by progressive hair loss confined to the scalp. This disorder occurs at similar rates in males and females. This process leads patients to almost complete baldness by the third decade. It can be inherited in an autosomal dominant or autosomal recessive manner. In almost 50% of the cases, the genetic etiology is not identified,^2^ even though many gene mutations have been associated. Dominant forms are related with mutations in CDSN, APCDD1 or SNRPE, while recessive forms are linked with biallelic mutations in LSS, KRT25, LPAR6, LIPH or DSG4.^4^ Few treatments have been described in the literature to be effective for this condition; however, our patient responded positively to oral minoxidil. Considering that small hair shaft diameter and high telogen/anagen ratio are the main factors responsible for hypotrichosis, it is reasonable to consider minoxidil as beneficial.^4^

Moreover, some articles document the efficacy of oral treatment with oral minoxidil and growth factors specifically for our patient's type of mutation.^4^ In conclusion, oral minoxidil can be used as a treatment to improve hair density and thickness in congenital hypotrichosis.

## AUTHOR CONTRIBUTIONS

Fabrizio Martora, Gabriella Fabbrocini, Teresa Battista, Sonia Ocampo‐Garza, and Marina Vastarella performed the research. Fabrizio Martora, Teresa Battista, and Gabriella Fabbrocini designed the research study. Angela Patri and Gabriella Fabbrocini contributed essential reagents or tools. Fabrizio Martora, Paola Nappa, and Mariateresa Cantelli analyzed the data. Fabrizio Martora and Teresa Battista wrote the paper.

## CONFLICT OF INTEREST

Gabriella Fabbrocini acted as a speaker or consultant for Abbvie, Amgen, Eli Lilly, Janssen, Leo‐Pharma, Almyrall, Novartis, and UCB. None of the contributing authors has any conflict of interest, including specific financial interests of relationships and affiliation relevant to the subject matter or discussed materials in the manuscript.

## Data Availability

Data sharing not applicable to this article as no datasets were generated or analyzed during the current study.
